# 3D Printed Contact Lenses

**DOI:** 10.1021/acsbiomaterials.0c01470

**Published:** 2021-01-19

**Authors:** Fahad Alam, Mohamed Elsherif, Bader AlQattan, Ahmed Salih, Sung Mun Lee, Ali K. Yetisen, Seongjun Park, Haider Butt

**Affiliations:** † Department of Mechanical Engineering, Khalifa University of Science and Technology, P.O. Box 127788, Abu Dhabi, United Arab Emirates; ‡ Department of Biomedical Engineering, Khalifa University, Abu Dhabi, P.O. Box 127788, United Arab Emirates; § Department of Chemical Engineering, Imperial College London, London SW7 2AZ, U.K.; ∥ Department of Bio and Brain Engineering, Korea Advanced Institute of Science and Technology (KAIST), Daejeon 34141, Republic of Korea; ⊥ KAIST Institute for Health Science and Technology (KIHST), Korea Advanced Institute of Science and Technology (KAIST), Daejeon 34141, Republic of Korea

**Keywords:** additive manufacturing, contact lenses, laser printing, nanopatterning, sensing

## Abstract

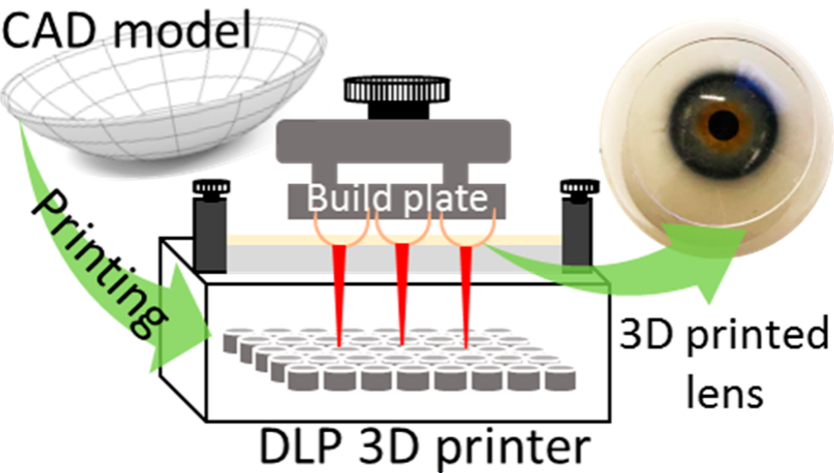

Although the manufacturing processes of contact lenses are well established, the use of additive manufacturing for their fabrication opens many new possibilities to explore. The current study demonstrates the fabrication of personalized smart contract lenses utilizing additive manufacturing. The study includes 3-dimensional (3D) modeling of contact lenses with the assistance of a computer aided designing tool based on standard commercial contact lens dimension, followed by the selection of the suitable materials and 3D printing of contact lenses. The 3D printing parameters were optimized to achieve the desired lens geometries, and a post processing treatment was performed to achieve a smooth surface finish. The study also presents functionalized contact lenses with built-in sensing abilities by utilizing microchannels at the contact lens edges. Tinted contact lenses were printed and nanopatterns were textured onto the contact lens surfaces through holographic laser ablation. 3D printed contact lenses have advantages over conventional contact lenses, offering customized ophthalmic devices and the capability to integrate with optical sensors for diagnostics.

## Introduction

The demand of noninvasive medical devises for the health monitoring requires development of medical devices with various sensing abilities.^1−5^ Contact lenses, one of the most widely used wearable devices, are utilized for vision correction and for cosmetics purposes.^6^ Nowadays, smart contact lenses are under development to be capable of monitoring various health conditions, such as dry eye disease, intraocular eye pressure, and glucose levels.^7−12^ Advantageously, the contact lens directly interacts with the tears, which contain substantial information regarding ocular complications and blood biomarkers.^13^ This information can be utilized for medical diagnosis and delivering targeted drugs. Nanostructures on the surface of contact lenses can be used as optical transducers; however, the integration of nanopatterns within the contact lens media is a huge challenge.^14^

Contact lenses are generally manufactured via spin-casting, molding, and lathe machining process.^15−18^ Spin-casting and molding are simple processes, where molds are required, and the anterior and posterior surfaces of the contact lens are formed in one step. In the molding process, the molds exhibit two surfaces that define the anterior and posterior surfaces; whereas in the spin-casting method, the anterior surface comes from the mold surface, and the posterior surface is generated by spinning the liquid monomer resin. Both spin-casting and molding processes are cost-effective, but they have the issue of adherence to the mold surface leading to additional post-process treatments.^19,20^ The lathe machining process involves the formation of anterior and posterior surfaces by cutting them from the button of the lens material. Lathe machining is a costly, time-consuming process, and is limited in terms of the designed geometries.^21,22^ All these processes are limited to some extent in terms of the required materials, the degree of design freedom, and the complex geometries. For manufacturing of customized, functionalized, and smart contact lenses—additive manufacturing (AM) techniques may be superior.

The additive manufacturing, also known as 3D printing technique, is the most emerging technology in the manufacturing field, especially in manufacturing of biomedical devices^23^ because of its control over dimensions with the help of a computer aided design (CAD) model of the object piled up into a 3D architecture with high accuracy.^24^ The method is attractive because it is time and cost-effective as well requires very little post processing of the printed objects. As compared to any other conventional manufacturing methods, AM is accurate in duplication of objects, and multiple objects can be manufactured at the same time. In recent years, AM has been well adopted and accepted globally as a manufacturing platform because of its improved 3D outputs and flexibility in terms of materials.^25−27^

To date, the 3D printed contact lenses are explored in few studies available in the literature.^28−30^ There are various types of 3D printing techniques being utilized for the manufacturing of optical devices,^31^ such as selective laser sintering (SLS),^32^ fused deposition modeling (FDM),^33,34^ photocuring stereoscopic printing, stereolithography apparatus (SLA),^35^ and digital light printing (DLP).^36^ Among these techniques, FDM is limited because of the low transparency of the 3D printed objects due to their thick printed layers (>0.1 mm) and the voids formed between the layers. Similarly, SLS is limited because of the necessity for post processing treatments, such as polishing, grinding, and deburring, which consumes a lot of extra time. In addition to the need for time-consuming processes, post processing is not possible for most of objects because of the complex shapes and the minute sizes of the optical devices manufactured. Light-curing-based 3D printing techniques (i.e., SLA and DLP) are usually preferable in manufacturing of optical devices, owing to the high resolution of the printing and the minimal thickness of the printable layers.

In the current work, the contact lenses are manufactured using DLP 3D printing technique using a transparent resin. The printing parameters were optimized to achieve acceptable transmittance levels for the lenses with adequate mechanical properties. Contact lenses with various geometrical features (microchannels) were also manufactured to portray the process’ versatility. Furthermore, tinted contact lenses were manufactured with acceptable transmittance properties. Additionally, nanopatterns were textured on the surface of the contact lenses with the help of direct laser interference patterning (DLIP). The samples were characterized by means of microstructural, mechanical, physical, and optical parameters to assess the potential of the selected manufacturing process and material.

## Experimental Section

### Materials

Asiga DentaClear Resin (transparent resin; ASIGA, Alexandria, NSW, Australia), a mixture of methacrylate and diphenyl (2,4,6-trimethylbenzoyl) phosphine oxide (the details of all chemical ingredients are given in the Supporting Information), isopropyl alcohol (Merk, Darmstadt, Germany), food colors (Foster Clark Products, Ltd., San Gwann SGN 3000, Malta EU), smooth surface plastic films (PVC, transparent films, commercially available), and deionized (DI) water were used.

### Preparation of 3D CAD Model of the Contact Lenses

The 3D models of the contact lens and flat discs were developed by SOLIDWORKS, a computer-aided designing (CAD) tool. The dimensions of the contact lens were obtained from the literature and utilized to design the model. The microchannels of three different dimensions were created from the CAD design, the images of the CAD model of the printed microchannels are shown in the Supporting Information (SI). The stereolithography (.stl) files were prepared which is then converted into 3D printer readable file.

### 3D Printing Procedure

**Figure 1:**
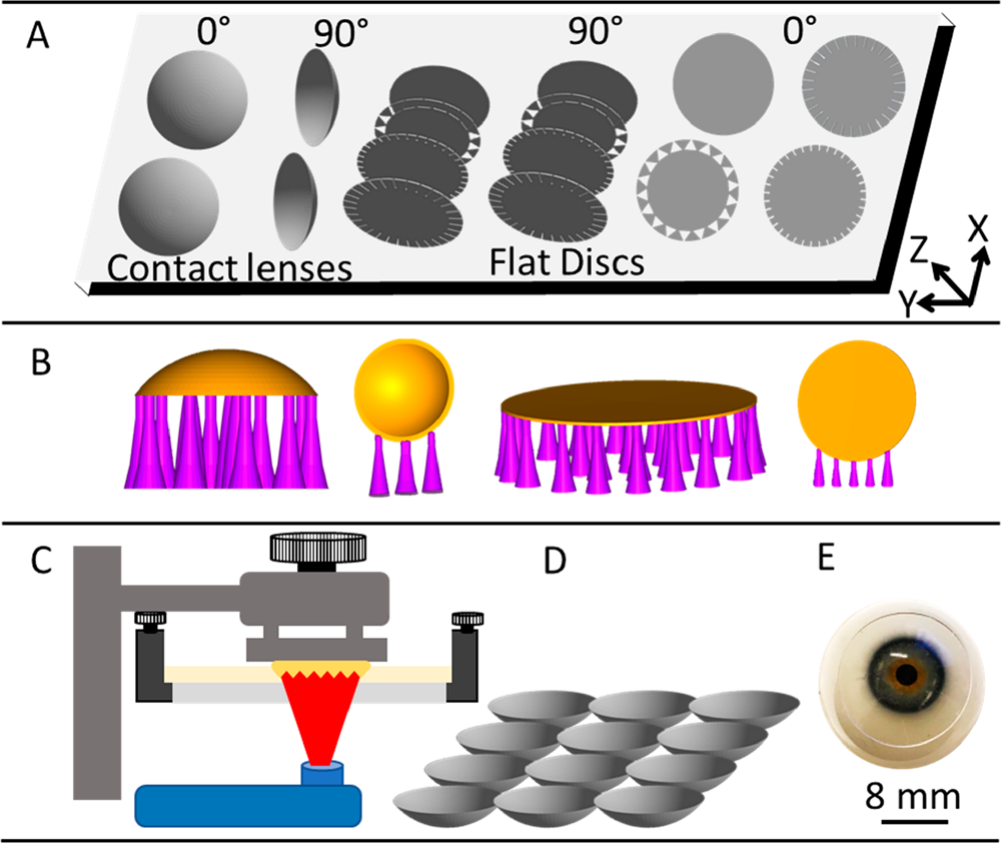
Schematic representation of the processes involved in DLP 3D printing of contact lenses. (A) Production of the CAD model of the lens, (B) preparation of 3D printer readable files with appropriate supports and two printing orientations, (C) DLP 3D printing, (D) removal and cleaning of the prepared lenses, and (E) end user application of the lenses.

The process of 3D printing of contact lenses was carried out using a digital light printing-based 3D printer (Wanhao D7 Plus, Jinhua, Zhejiang, China). The fabrication steps involved in the whole process are shown in Figure 1. The 3D model developed by CAD tool in the form of .stl file is displayed in Figure 1A. The supports of the printed object were added with the help of WanhaoD7workshop slicing tool, as shown in Figure 1B, and the printing parameters were optimized and set for all the samples. The details of the printing parameters are presented in Table SI1. Multiple samples were spread all over the print bed (Figure 1A). Finally, the file was saved in the .cws format and supplied to the printer to initiate printing. The process of DLP 3D printing is depicted schematically in Figure 1C, showing the light source, resin tray, and print bed. The curing of the liquid monomer resin was carried out by the UV light sources. After printing, the samples were removed (Figure 1D) from the print bed, and the supports were removed and washed with the iso-propyl alcohol (IPA) solution. The washing was done twice to remove any uncured resin and deposits to obtain the final product shown on an eye model, Figure 1E.

To achieve the desired level of optical transmittance from the 3D printed lens, post printing treatment was utilized. Dip coating (30 s), followed by a curing process (2 min), was adopted for this purpose. The printed samples were removed from the print bed and washed with IPA solution. The washed samples were dipped in the liquid monomer resin for 1 min and then cured in UV chamber for 30 s. After curing, again samples were washed with IPA to remove any uncured resin.

### Characterization of Lens Material

**Figure 2:**
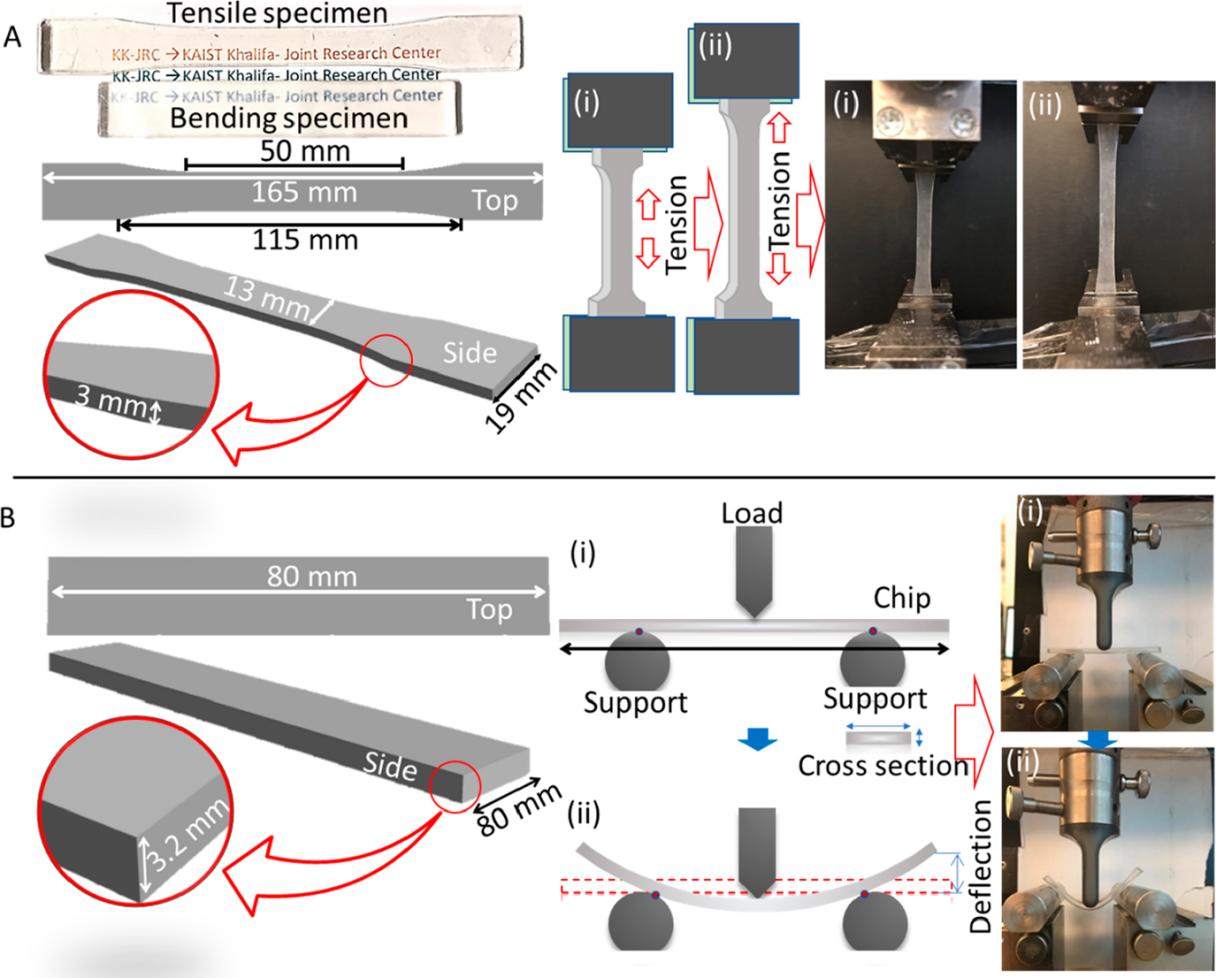
Mechanical testing of the lens material. (A) Samples of tension test and the tensile test setup showing the schematics representation of the dogbone samples and the clamps of the UTM holding the dogbone samples. The samples before and while in tension are shown in the photographs. (B) Three-point bending test of the material. The schematics are showing the test specimen and the process of 3-point bending. The samples before and after bending are shown in photographs as indicated by arrows.

The physical and structural properties of the manufactured lenses and discs were characterized. The surface topography of the manufactured samples was observed using scanning electron microscopy (SEM, The FEI Nova NanoSEM 650) with an accelerating voltage of 15 kV and a working distance of 5 mm. A thin layer of Ag/Pt (10 nm) was deposited onto the surface by the DC sputtering system for SEM imaging. The surface roughness of the samples was measured using atomic force microscopy (AFM). The scanning of the surface was done using a V-shaped AFM cantilever (Bruker NP-10 Camarillo, CA USA). The data analysis was performed using Gwyddion; a data visualization and analysis software. X-ray diffraction (XRD) analysis was performed on a 3D printed flat disc to check the crystallinity nature of the lens material. Cu Kα radiation (λ = 1.54 Å) operated at 45 kV and 30 mA was used as X-ray source (using XRD PAN analytical Empyrean diffractometer) with a scan speed of 2.4 deg/min and a step size of 0.02° with 2θ ranging from 10° to 60°. The wettability of the materials was measured using a contact angle homemade setup. The sessile drop method was opted for this study as it is fast and easy method to measure contact angle in static mode. Drops of 5 and 15 μL were placed on the surface of the discs. A total of 9 drops (3 drops on 3 sample copies) were placed and the average results is reported. The mechanical performance of the 3D printed samples was characterized by performing compression and bending test. Uniaxial quasi-static tensile tests were conducted as per ASTM D638 standard (Figure 2A)^37^ using a Zwick-Roell Z005 universal testing machine fitted with a 2.5 kN load cell, at a constant crosshead speed of 1 mm/min at ambient temperature (∼24 °C). The modulus of elasticity, tensile strength and elongation were calculated from the stress–strain curve derived from load–displacement curves obtained from the tensile test. The cross-sectional area and gage length were utilized to calculate the stress and the strain at each point.

The three-point bending test was performed following the ASTM D790 standard^38^ on a flat rectangular bar of 80 × 12.7 × 3.2 mm^3^ (Figure 2B). The span between the supports was fixed at 50 mm, and the test were performed at a speed of 2 mm min^–1^. The force and elongation were recorded and utilized to calculate the elastic modulus and bending strength. Since the contact lens is always wet due to direct contact with eye tears, the mechanical test was performed on wet samples. For this purpose, the 3D printed specimens were immersed in water for 24 h, and then, the tests were performed. To ensure moisture conditions, water was sprayed on the samples during the test.

The flexural stress (*σ*_f_), flexural strain (*ε*_f_), and the modulus of elasticity in bending (*E*_B_) were determined using following equations:
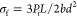
1
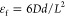
2
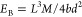
3where *P*_*i*_ is the load at a point on the load–displacement curve, *L* is the span between the support, *b* is the specimen width, *d* is the specimen depth, and *D* is the maximum defection of the center of the specimen. The dimensions of the test specimens are shown in Figure 2.

### 3D Printing of Tinted Contact Lenses

To develop the colored contact lenses, nontoxic and food grade colors were utilized. In the process, the food colors were mixed with liquid monomer resin using a magnetic stirrer for 15 min. After proper mixing, the colored resin monomer was filled in the 3D printer tray, and the printing process was carried out. After printing, the samples were washed with IPA, followed by water bath to remove any uncured resin and loose color material. The same procedure was followed to manufacture various colored lenses. To access the feasibility with multiple colors, four colors, that is, blue, green, red, and yellow were utilized with same amount of dying. All the colors were added in the amount of 2 vol % to achieve the coloration without affecting much the transmittance of the lenses (Figure 9).

### Nanopatterning of the Surface of Contact Lenses

The nanopattern on the surface of the 3D printed contact lens was developed via holographic laser ablation method. The laser ablation process was carried out via direct laser interference patterning (DLIP) method in holographic Denisyuk reflection mode. In this process, to facilitate the interaction between the laser beams and the lens material a black color dye was utilized. The whole process of integration of the nanopattern on the lens material was done by carrying out the following steps. In the first step, the disc used (diameter = 16 mm, thickness = 200 μm) was cleaned with IPA and placed on a glass slide. In the next step, the synthetic black dye was applied to the surface of the disc. The thickness of the dye was selected based on the values obtained from the previous work of the research group.^14^ The holographic nanopattern was generated because of the interference between the incident and reflected laser beams. Upon exposure of laser the ablative interference fringes were developed leaving a nanopattern on the surface of the lens. Finally, a one-dimensional (ID) nanopattern was generated on the surface of the 3D printed contact lens. The nanopattern produced on the surface of the samples was observed under SEM.

## Results and Discussion

**Figure 3:**
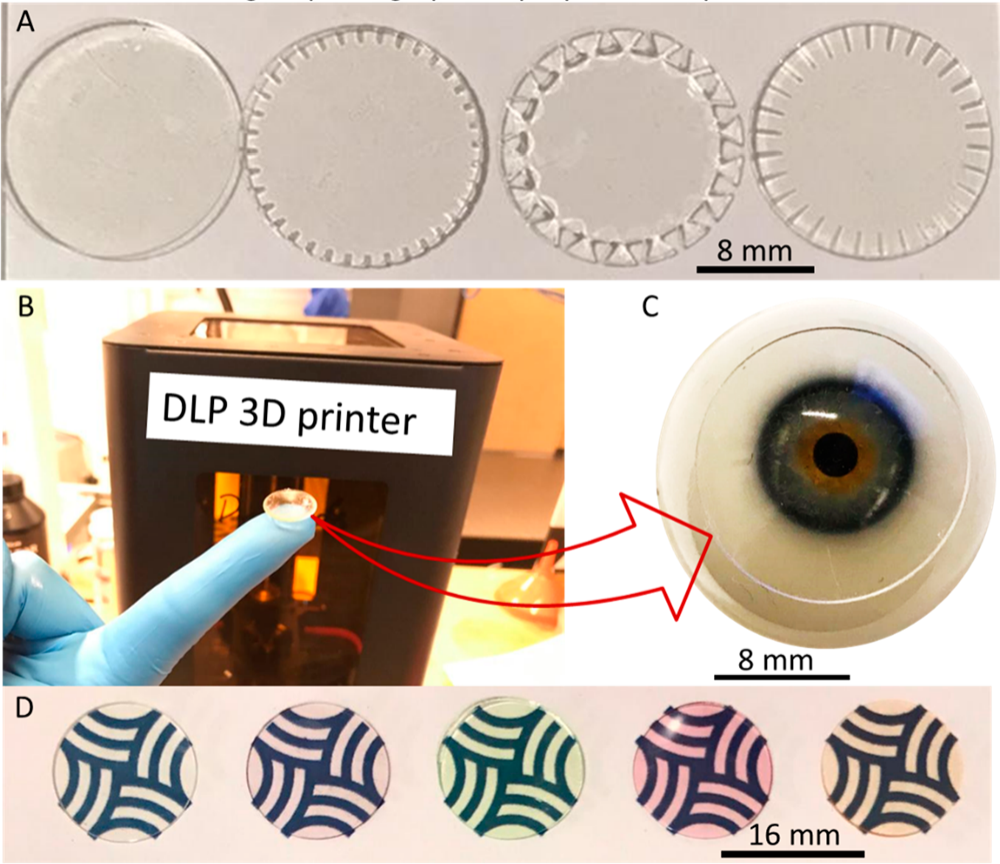
Digital photographs of the lenses and disc fabricated by 3D printing. (A) Flat disc with different integrated microchannel designs. (B) Digital photographs of a curved lens on the tip of a finger. (C) Contact lenses on an eye model showing the transparency of the lens. (D) Digital photographs of the plain and tinted contact (left to right) lenses showing the feasibility of the manufacturing process and the optical visibility.

The 3D printed contact lenses and discs are shown in Figure 3. Flat discs with integrated edge microchannels are shown in Figure 3A. As clearly visible from the photo that with the 3D technology, it is possible to manufacture contact lenses of desired dimensions and complex geometrical microchannels at the edges because of the feasibility of CAD designing. Three different geometries were demonstrated to prove the ability of the current process for the manufacturing of smart multifunctional lenses. These microchannel can be exploited to act as optical transducers^39^ by observing the change in the microchannel geometries with the help of images captured from smart phones or any other camera. For example, dry eye sensing can be performed by monitoring the spacing between the channels.^40^ The contact lens manufactured using 3D printing technique is shown in Figure 3B and C, and it can be observed that the lenses cover the artificial eyeball with appropriate optical transparency.

**Figure 4:**
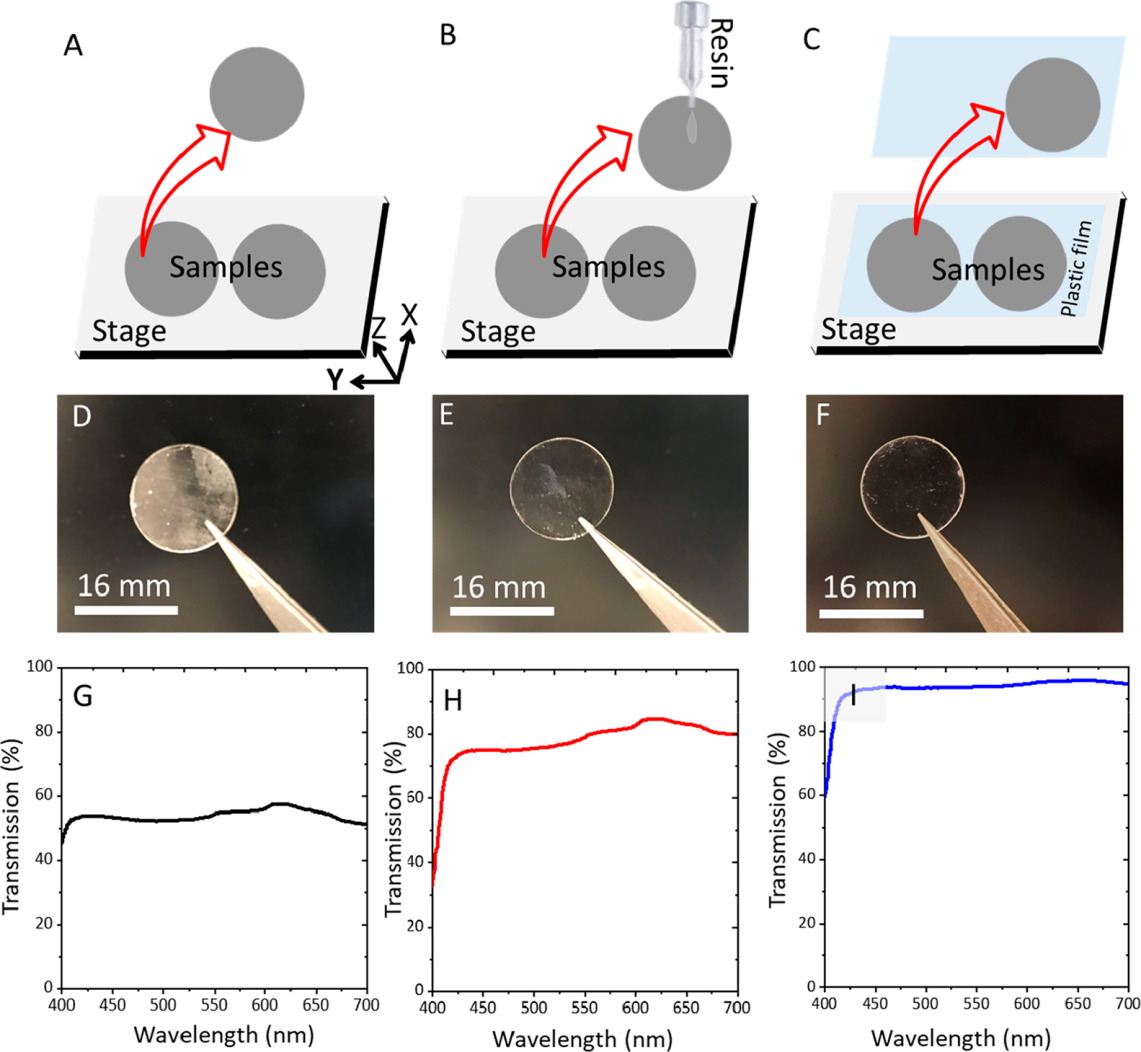
Three stages of optimization of the samples while printing. (A) Printing directly on the print bed without post processing. (B) Printing on print bed, followed by resin coating, and (c) printing on a PVC thin film attached on the print bed. Photographs of the samples obtained via (D) the print bed, (E) resin coating and (F) from the PVC film. Similarly, the transmittance spectra with respect to wavelength are shown for samples (G) obtained directly from print bed, (H) resin-coated sample, and (I) obtained from PVC film.

The tinted discs manufactured using the 3D printer are shown in Figure 3D. The digital photographs of the discs were taken on Khalifa University’s logo to present the different color shades and transparency. Colors of the lenses are clearly visible and intact even after storing them in deionization (DI) water overnight. The discs initially obtained from the 3D printing process were showing a lower range of transmittance (∼50%) upon exposure to visible light. So, the optimization process^41^ was carried out to achieve the satisfactory level of transmittance, and the results are shown in Figure 4. The lower range of transmittance was due to the strong adherence of the samples with the print bed causing surface damaged during the removal process, as well as the poor surface finish of the print bed (Figure 4A). The rough surface of the print bed left its impression on one side of the lens, which scatters light resulted in poor normal transmittance (Figure 4D). Furthermore, the discs were quite thin, so their removal from the bed without damage was a big challenge. The samples were removed from the print bed by sharp blades, which also causes some dent and scratches to that lower surface of the discs and resulted in a lower transmittance (between 50% and 55% across the optical spectrum). In the second stage of optimization, post-processing treatment was adopted to improve the transmittance.^35^ The samples were coated with a thin layer of the monomer liquid resin after removal from the print bed (Figure 4B). This process successfully improved the transmittance by almost +30% as compared to the results without the post treatment. The level of transmittance reached ∼80% (Figure 4E). Because of the resin coating, the scratches and dents were filled with the uncured resin, which hardened upon exposure to UV light. This way a smoother surface was obtained as compared to the untreated one. The third approach that was opted to improve the transmittance included the application of a thin film of PVC plastic on top of the print bed (Figure 4C). This improved the surface finish of the printed samples and improved the overall optical transmittance (Figure 4F). It can be clearly observed from the digital photographs of all three samples shown in Figure 3D–F that the samples printed on PVC plastic film (Figure 4F) have the most clarity and exhibited light transmission about 90%, that is, ∼40% more than that of the first approach and ∼10% more than that of second approach. The advantage of printing on a PVC plastic film was that it made it very easy to remove the samples from the print bed. The smooth surface of the PVC film and easy removal procedure allowed the lenses to achieve a high level of transmittance.

**Figure 5:**
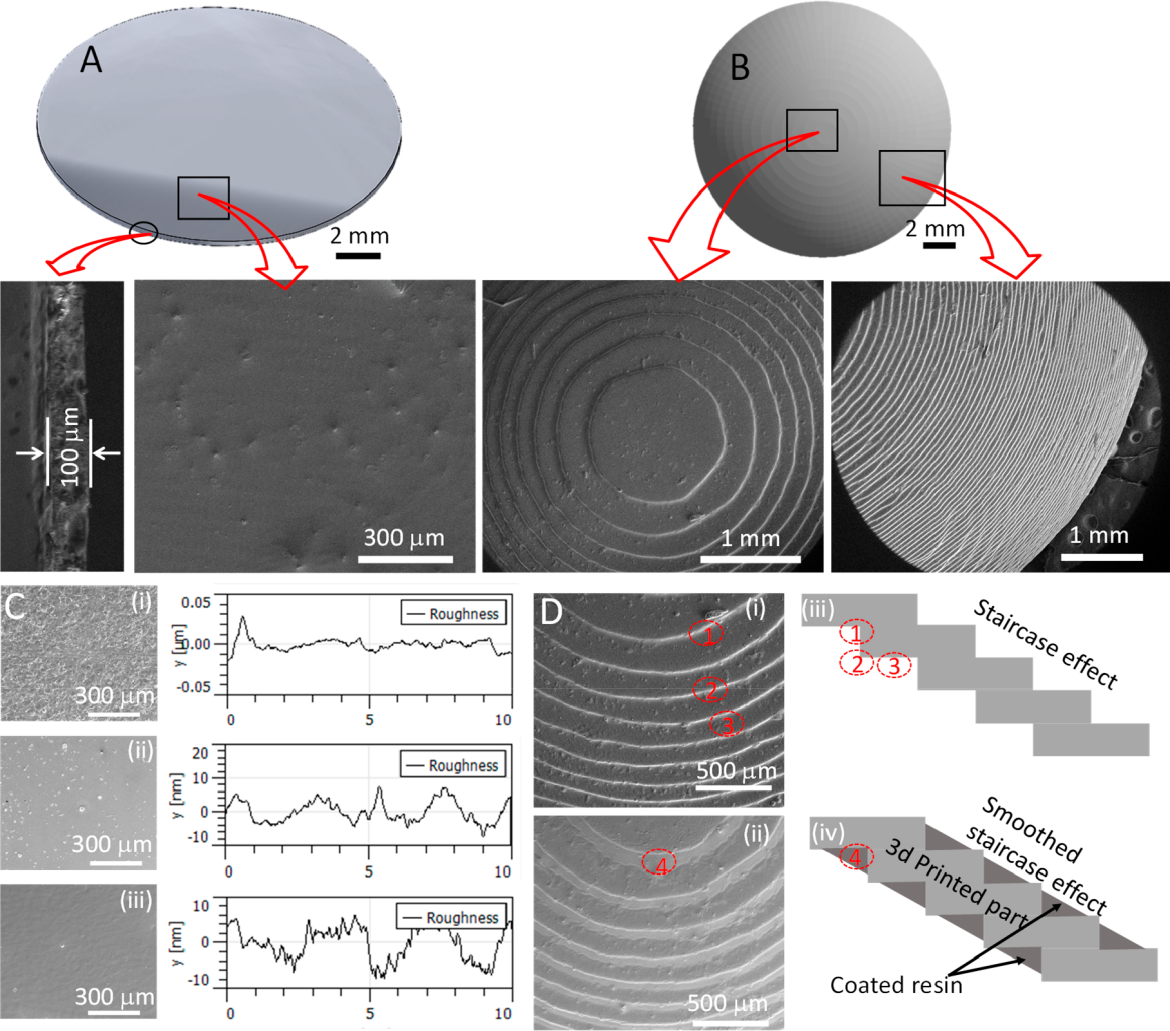
Different developmental stages in optimization of lens and discs with adequate transmittance required for a contact lens. (A) SEM micrograph of the flat disc, the top surface and cross section are highlighted with the red arrows. (B) SEM micrograph of the contact lens, the top surface and side wall of the lens are highlighted with the red arrows. (C) SEM micrograph of the flat disc manufactured with 3D printing showing the improvement in the surface roughness by opting the 3 different approach (i) directly print on print bed, (ii) post-print resin-coated, and (iii) printed on PVC plastic film. The corresponding surface topography obtained from AFM are shown in the right side. (D) SEM micrographs of the contact lens before (i) and after (ii) dip coating after 3D printing and the effect of dip coating is depicted with the help of schematics (iii and iv).

The surface roughness of the samples obtained from all these three approaches were measured using AFM and the results are shown in Figure 5C. The average roughness (*R*_a_) was ∼50 nm for first approach, where it is reduced to ∼25 and ∼10 nm for second and third approach to improve the transmittance level. The samples were further observed under SEM to visualize the surface morphology at a larger scale and micrographs (Figure 5Ci–iii). The manufactured flat disc and contact lens were observed under SEM and the results are shown in micrographs in Figure 5. The thickness was confirmed for flat disc by observing cross-section of the discs, and it was found to be ∼100 μm as shown in Figure 5A. The top surface of the disc was observed, which showed a smooth surface indicating the good quality of printer and parameters selected in this research. Similarly, the contact lens was also observed in SEM from the top and from the sides. Staircase effect was very clear from the micrographs of Figure 5B. The staircase effect was reduced by the post manufacturing treatment process,^42,43^ that is, dip coating (30 s) with monomer liquid resin followed by UV curing (2 min). The lens was again observed under SEM after treatment and the results are shown in Figure 5Di and ii. Schematically, the process of staircase effect reduction is shown in Figure 5Diii and iv. As indicated in the scheme, the stairs corners were filled by the liquid monomer resin, which is then solidified with UV light. The SEM images also confirms that the process was successful as a clear difference between uncoated (Figure 5Di) and coated samples (Figure 5Di and ii) are visible, which indicates the corners were filled with resin and a smoother surface was produced.

**Figure 6:**
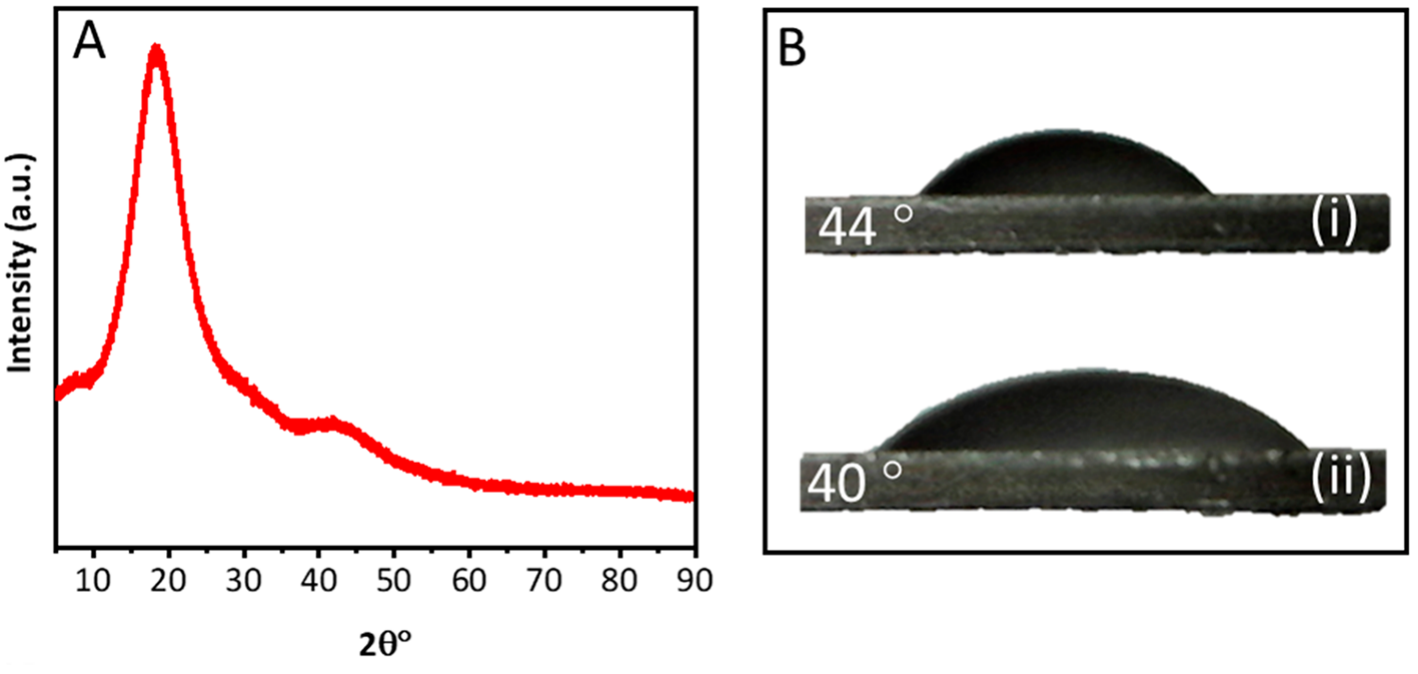
Material propertied of the lens material. (A) XRD spectra and (B) surface wettability: (i) 5 μL droplet and (ii) 15 μL water droplet.

The material properties were characterized by means of physical, chemical, and mechanical analysis, and the results are presented in Figure 6. The characterization of the crystallite nature of the 3D printed lens was carried out and the corresponding spectra is shown in Figure 6A. Only a hump at ∼20° (2θ°) and no other sharp peak was observed, which indicated the amorphous nature of the material.^44^ The surface wettability of the lens materials was performed on flat thin discs and digital images of the sessile droplet is shown in Figure 6B. The water contact angle was measured and found to be in the range of 40–44°. This confirms the hydrophilic nature of the manufactured samples.^45^

**Figure 7:**
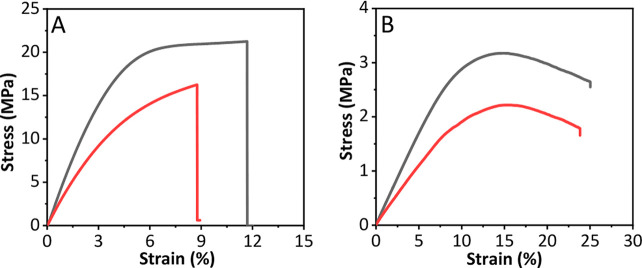
(A) Stress–strain curve obtained after tension test and (B) stress–strain cure obtained after 3-point bending test.

The mechanical properties are critical for contact lenses for handling, durability, and comfort. The mechanical properties were analyzed in dry and wet conditions, and the stress–strain curve was used to quantify the tensile and flexural mechanical properties. The representative curve obtained from tensile tests are shown in Figure 7A and curve obtained from 3-point bending test is shown in Figure 7B. In both tests, the mechanical pores were decreased in wet samples. The Young’s moduli were calculated from the initial linear region, viscoelastic portion, of the curve, and the results are summarized in Table 1.

**Table 1:** Mechanical Properties of the Lens Material Obtained from the Tensile and 3-Point Bending Test^a^

	dry	wet
tensile properties		
modulus (MPa)	5.56 ± 0.13	3.78 ± 0.08
strength (MPa)	21.01 ± 0.14	16.30 ± 0.22
elongation (%)	11.60 ± 0.05	8.7 ± 0.11
flexural properties		
flexural modulus (MPa)	8.08 ± 0.22	7.61 ± 0.36
strength (MPa)	43.03 ± 2.16	39.57 ± 3.56
deflection (%)	14.13 ± 1.87	14.38 ± 2.56

a The tests were performed on the dry and wet samples.

Mechanical properties were found to decrease after immersion in water for 24 h. Both the tensile and the bending mechanical properties were lower for the wet samples.^46^ As the thicker samples manufactured by 3D printing contains multiple layers. So, the decrease in the mechanical properties after immersion in water could be because the interfacial adhesion between the printed layers weakens the layer bonds.^47^ Water molecules in the wet environments could penetrate in between the layers through micropores or microcracks or and could reduce the interfacial adhesion between the consecutive layers.

**Figure 8:**
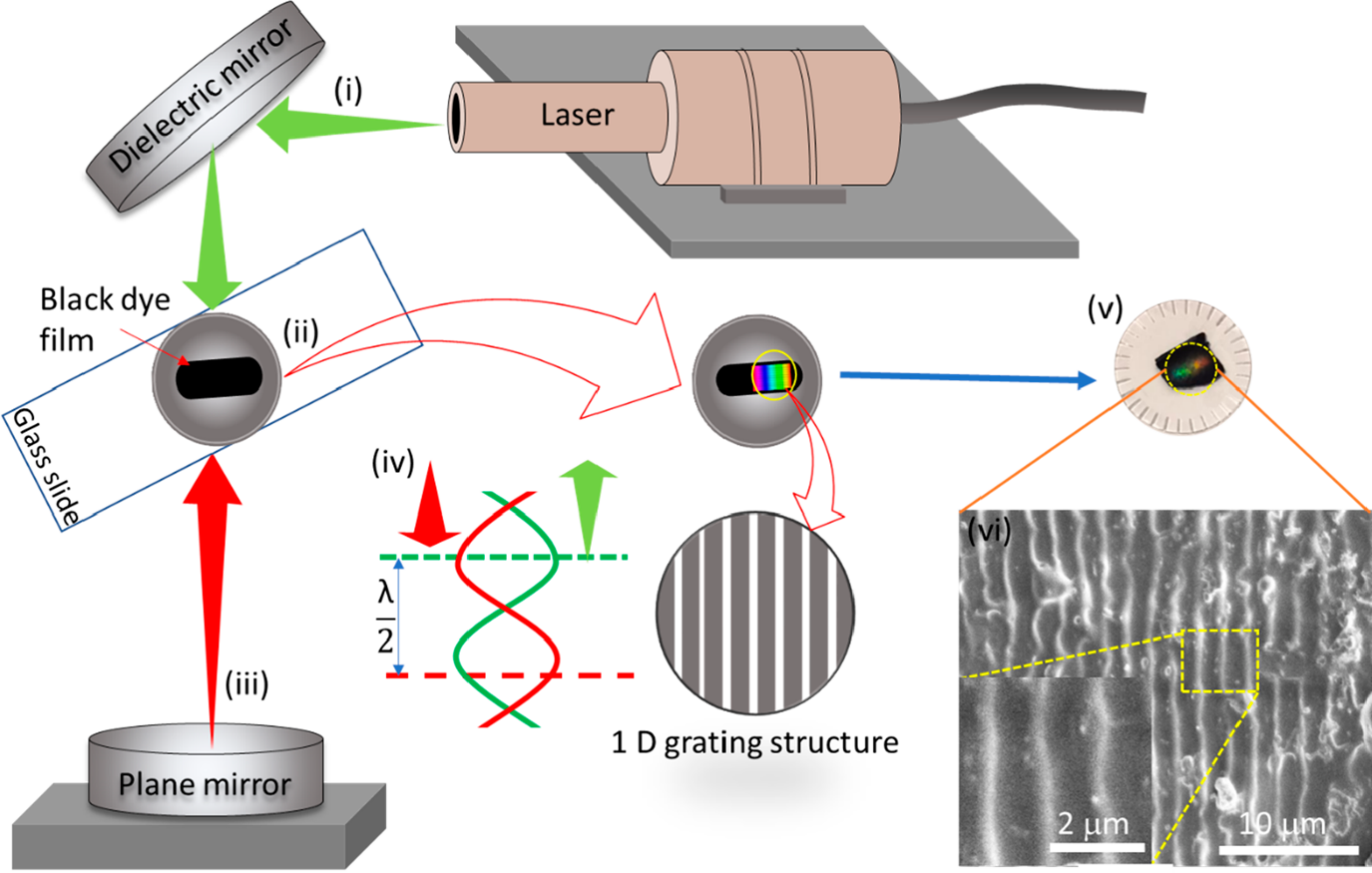
Fabrication of the nanopattern on the surface of contact lens using a DLIP in Denisyuk reflection mode. Nd:YAG laser beam (1064 nm focused beam) was used to create the nanopattern on the surface of the lens. (i) The laser beam was guided by a mirror and (ii) passed through the dyed contact lens. (iii) The laser was reflected back from a plane mirror placed perpendicular to the incident beam. (iv) The ablation process created a 1D grating structure, which displayed a rainbow holographic effect due to diffraction. (v) The digital photograph of the hologram is shown on the contact lens manufactured via 3D printing. (vi) The SEM micrographs show the nanopattern.

The nanopattern developed on the surface of the manufactured lens using DLIP method in Denisyuk reflection mode is shown in the Figure 8. The laser beam was guided by a mirror directed to incident on the recoding medium, that is, synthetic dye, deposited on the surface of the lens. The laser beam was reflected back owing to hitting a plane mirror located below the lens to facilitate the laser ablation process on the selected regions. The standing wave was generated because of the interference of incident and reflected wave allowing the formation of ablated and nonablated regions on the dye, giving a 1D (one-dimensional) grating structure.

Because of the high energy in the constructive interference regions, the nanogrooves were produced^48^ on the surface of the contact lens as shown in the Figure 8. A high power interference beam is produced when incident beam and reflected beam interacted and resulted in ablation.^49^ The grating spacing depend on the angle of exposure. For example, a grating spacing of 925 nm can be created at an exposure angle of 35° from the horizontal planar. The grating spacing can be calculated theoretically with the help of eq 4(14)
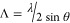
4where Λ is the grating spacing, λ is the wavelength, and θ is the exposure angle (tilt angle) of the samples with the horizontal plane. The grating spacing observed from SEM micrographs was found to be around 960 nm as shown in Figure 8vi. The holographic nanopattern integrated on the contact lenses can be utilized as a transducer to sense electrolytes concentration in the tears, which will reflect the physiological state of the eye. Sensing the electrolyte concentration in tears could give a lead into detection of early decease state of the eye.^14^

**Figure 9:**
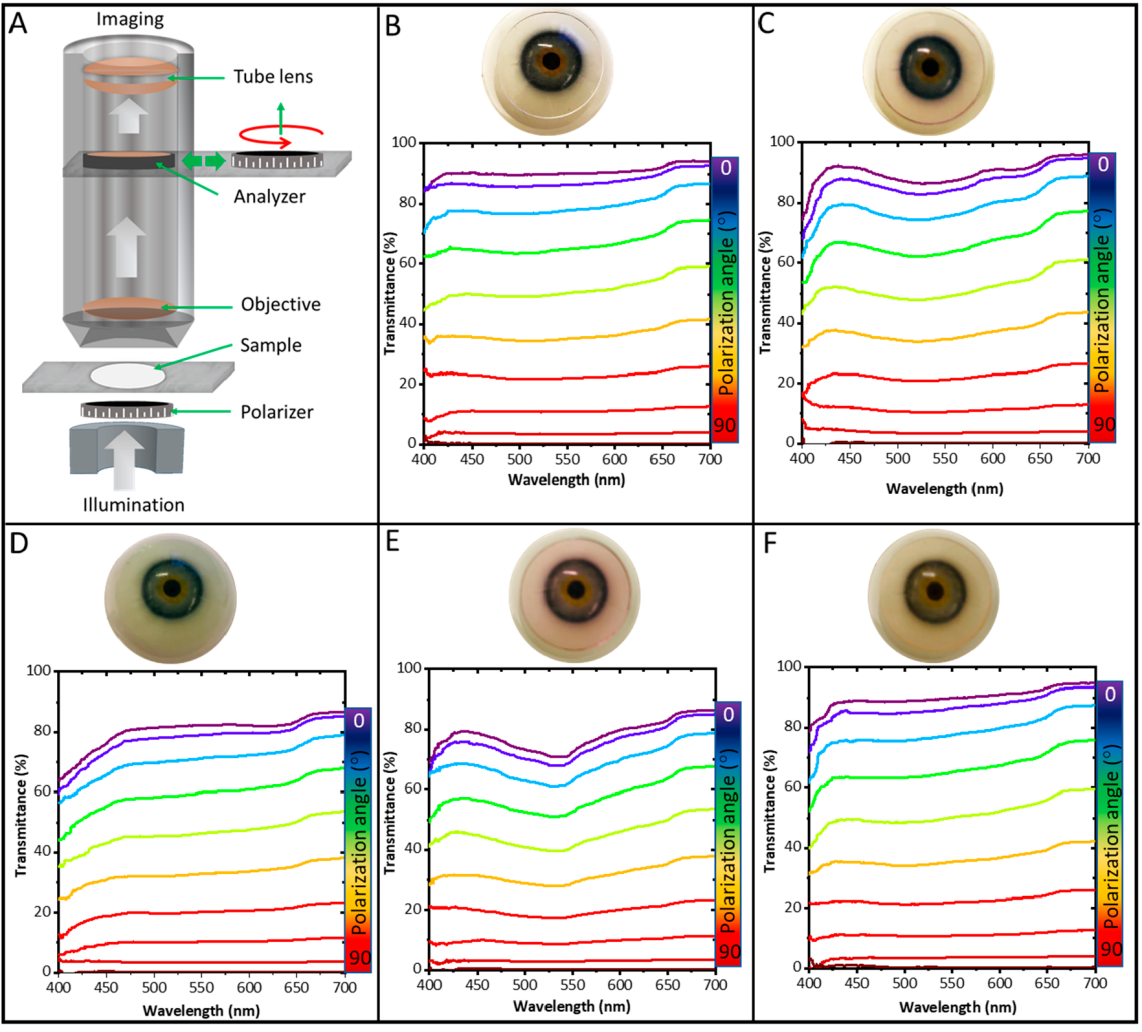
Optical polarization spectroscopy of the dyed contact lenses manufactured via 3D printing. (A) Schematic representation of the setup used for the measurement of the polarization spectra. The transmission of polarized and unpolarized light with respect to wavelength was recorded at various polarization angle. The plots are shown along with their corresponding digital images of the dyed contact lenses: (B) no color, (C) blue, (D) green, (E) red, and (F) yellow color.

The tinted contact lenses were successfully fabricated using food grade colors in the liquid monomer resin via 3D printing and samples are shown in Figure 9. The digital photographs of the manufactured lenses are shown on an eye model for each color. The food colors were mixed in the liquid resin itself before the 3D printing step, which resulted in the embedding of the color into the polymer during polymerization, and the colors were stable as confirmed from vigorous washing in ethanol and water. Figure 9A shows the schematic representation of the setup used for measuring the polarization dependent transmission spectra. Optical polarization spectroscopy was performed on the tinted samples, and the transmission of polarized and unpolarized light with respect to wavelength was recorded at various polarization angles. No effect of color incorporation on the polarization of light was observed, and the same trend was observed for all samples. The light transmission obtained from the samples suggest that the additive manufacturing is a suitable processing method for plane and tinted contact lenses. However, the current work is focused mainly on the manufacturing process, and the performance toward the biosensing, and other capabilities of the 3D printed smart contact lenses are projected to be future studies.

## Conclusions and Future Aspects

In the current work, contact lenses were successfully manufactured via a DLP-based 3D printing method. Commercially available transparent resin monomer was used to manufacture the contact lenses. The material was characterized and found to have suitable properties required for a contact lens. The optimal 3D printed samples exhibited above 90% of light transmission. The mechanical properties of the samples showed a tensile Young’s moduli in dry and wet conditions of 5.56 ± 0.13 and 3.78 ± 0.08 MPa, respectively; whereas, the flexural modulus was 8.08 ± 0.22 and 7.61 ± 0.36 MPa for dry and wet samples, respectively, which is sufficient for handling and durability of the contact lenses. The feasibility of the 3D printing process was also explored to create the microchannels at the edges of the contact that can be potential transducer for sensing various ocular parameters. Furthermore, the colored contact lenses were successfully manufactured with the 3D printing method without affecting the light transmission. The tinted contact lenses with different dyes are potential candidate for giving unnatural color to the eyes and also can be a solution for the correcting colorblindness.^50^ A nanostructure pattern was also successfully integrated on the 3D printed contact lens with the help of laser ablation. The nanostructure generated on the surface of the contact lens can be utilized as a transducer for sensing ocular parameters. Additive manufacturing is one of the most suitable technique to manufacture the smart lenses and could be very much helpful in the area of biosensors smart wearable contact lenses. The future plan for this work is to explore the oxygen permeability of the manufactured lenses and also the sensing performance of microchannels and nanopatterns engraved on the surface of 3D printed contact lenses.

## Supporting Information Available

Printing parameters, CAD models, and surface topology (PDF)
