# Supplementary material for: 3D Printed Contact Lenses
Source: ACS Biomater Sci Eng. 2021 Jan 19;7(2):794–803. doi: 10.1021/acsbiomaterials.0c01470 (PMC8396802; doi:10.1021/acsbiomaterials.0c01470)
Supplement: Supplementary file 1 — ab0c01470_si_001.pdf [file ab0c01470_si_001.pdf]

SUPPORTING INFORMATION

3D Printed Contact Lenses

Fahad Alam[1,*], Mohamed Elsherif[1], Bader AlQattan[1], Ahmed Salih[1], Sungmun Lee[2], Ali K. Yetisen[3], Seongjun Park[4,5] and Haider Butt[1,*]

[1]Department of Mechanical Engineering, Khalifa University of Science and Technology, P.O. Box 127788, Abu Dhabi, United Arab Emirates

[2]Department of Biomedical Engineering, Khalifa University, Abu Dhabi, Po Box 127788, United Arab Emirates

[3]Department of Chemical Engineering, Imperial College London, London SW7 2AZ

[4]Department of Bio and Brain Engineering, Korea Advanced Institute of Science and Technology (KAIST), Daejeon 34141, Republic of Korea

[5]KAIST Institute for Health Science and Technology (KIHST), Korea Advanced Institute of Science and Technology (KAIST), Daejeon 34141, Republic of Korea

**E-mail: Fahad.Alam@ku.ac.ae (F.A.), haider.butt@ku.ac.ae (H.B.). Tel.:+971 2 401 8168*

Materials: *The chemical composition of the Asiga's Dentaclear resin: The resin is the mixture of following chemical ingredients:*

1. *7,7,9(or 7,9,9)trimethyl-4,13-dioxo3,14-dioxa-5,12diazahexadecane-1 16diyl bismethacrylate (10-20% concentration),*
2. *Tetrahydrofurfuryl methacrylate (10-25% concentration)*
3. Diphenyl(2,4,6- trimethylbenzoyl) phosphine oxide *(10-20% concentration).*

Table SI 1: DLP 3D printing parameters for manufacturing contact lenses.

**Printing parameters**	**Value**
Layer thickness	35 microns
Curing time	Burn in layers 80 s, Normal layers 75 s
Speed (Normal layers)	Lifting speed 40 mm/min, lowering speed 80 mm/min
Speed (slow selection layers)	Lifting speed 25 mm/min, lowering speed 40 mm/min
Wait after lift	1 s
Lift after print	6 mm
Support	Diameter; 0.3 mm, height; 4 mm

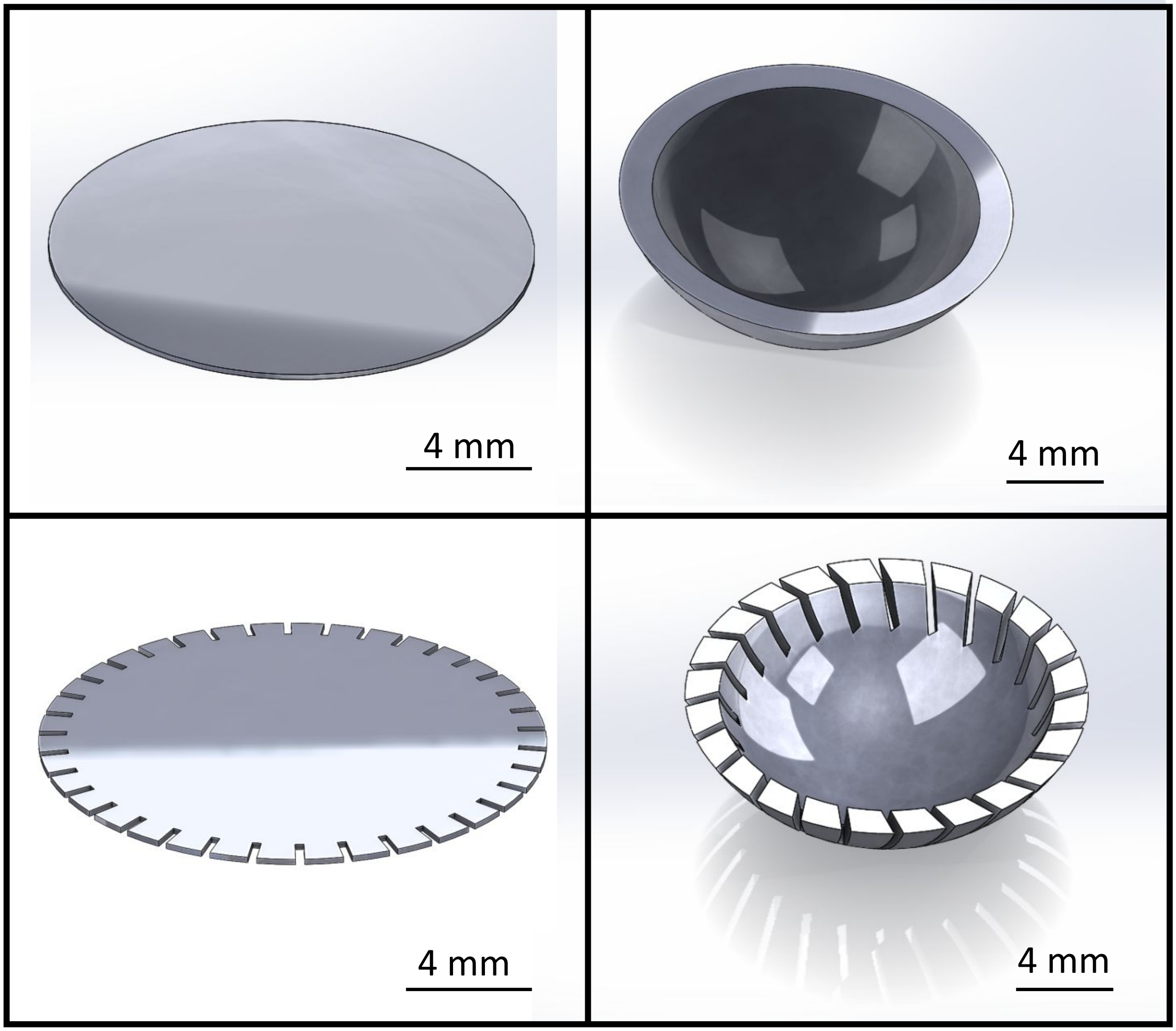

Figure SI 1. CAD modeling of lenses and discs

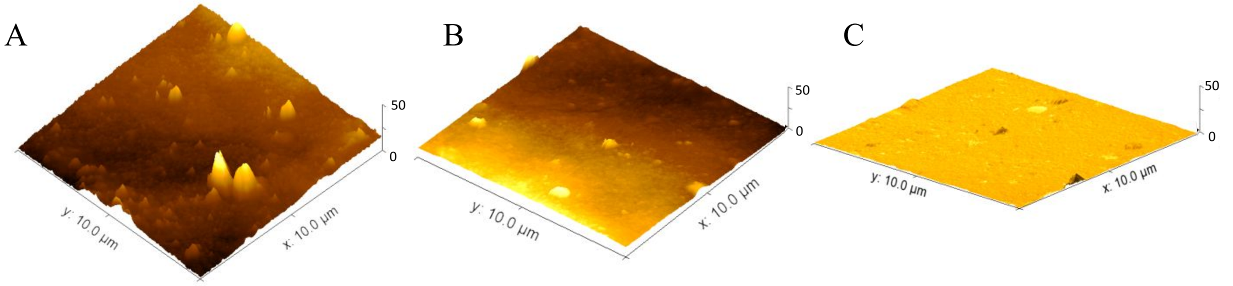

Figure SI 2. 3D surface topography of the discs taken from atomic force microscopy. (A) Sample printed directly on the print bed and no post processing were done. (B) Sample printed on print bed followed by resin coating and (c) sample printed on a PCV thin film attached on print bed.
